# Using traditional ecological knowledge to understand and adapt to climate and biodiversity change on the Pacific coast of North America

**DOI:** 10.1007/s13280-019-01218-6

**Published:** 2019-10-09

**Authors:** Victoria Rawn Wyllie de Echeverria, Thomas F. Thornton

**Affiliations:** 1 Environmental Change Institute, School of Geography and the Environment, South Parks Road, Oxford, OX1 3QY UK; 2 Linacre College, St. Cross Road, Oxford, OX1 6JA UK; 3 School of Arts and Sciences, University of Southeast Alaska, 11066 Auke Lake Way, Juneau, AK 99801 USA

**Keywords:** Adaptation, Biodiversity, Climate change, Ethnoecology, Local knowledge, Pacific Northwest Coast

## Abstract

We investigate the perceptions and impacts of climate change on 11 Indigenous communities in Northern British Columbia and Southeast Alaska. This coastal region constitutes an extremely dynamic and resilient social-ecological system where Indigenous Peoples have been adjusting to changing climate and biodiversity for millennia. The region is a bellwether for biodiversity changes in coastal, forest, and montane environments that link the arctic to more southerly latitudes on the Pacific coast. Ninety-six Elders and resource users were interviewed to record Traditional Ecological Knowledge (TEK) and observations regarding weather, landscape, and resource changes, especially as concerns what we term Cultural Keystone Indicator Species (CKIS), which provide a unique lens into the effects of environmental change. Our findings show that Indigenous residents of these communities are aware of significant environmental changes over their lifetimes, and an acceleration in changes over the last 15–20 years, not only in weather patterns, but also in the behaviour, distributions, and availability of important plants and animals. Within a broader ecological and social context of dwelling, we suggest ways this knowledge can assist communities in responding to future environmental changes using a range of place-based adaptation modes.

**Electronic supplementary material:**

The online version of this article (10.1007/s13280-019-01218-6) contains supplementary material, which is available to authorized users.

## Introduction

While the effects of climatic change are being felt by people around the world (Fatoric and Chelleri 2012), Indigenous Peoples are often living on the forefront of changes, and disproportionately impacted (Turner and Clifton 2009). Although a number of studies incorporating Indigenous knowledge of climate change have been conducted among especially vulnerable groups, the majority of this research is centred in the Arctic (e.g. Krupnik and Jolly 2002; Ford et al. 2006; Crate and Nuttall 2009; UNESCO 2009; Ford et al. 2010; Berkes 2012; Nakashima et al. 2013), or Small Island States (Barnett and Campbell 2010; Lazrus, 2012; Rudiak-Gould 2013). A small body of research in the Pacific Northwest examining TEK, oral history, and landscape perceptions, has touched on environmental change issues to some degree (Dauenhauer and Dauenhauer 1987; Cruikshank 2005; Fedje and Mathewes 2005; Thornton 2008; Turner 2014). However, our research aims to contribute to the even smaller body of literature from the Pacific Northwest that focuses specifically on how to integrate human perceptions of, and responses to, environmental change, especially the effects of climate change on biodiversity, and tie this into climate change responses and adaptations (Turner and Spalding 2013).

Coastal environments are known to be particularly sensitive to climatic change, as these ecosystems exist in delicate balance at the land-sea interface and have complex interactions between biological and geophysical changes (Moss 2005; Harley et al. 2006; Nicholls et al. 2007; Turner 2007; Glick et al. 2010; Fatoric and Chelleri 2012). Throughout these regions, changes often consist of rising sea levels, warming temperatures, decreasing pH, changing abundance and distribution of species, and unpredictable weather (Nicholls 2003; Moss 2005; Harley et al. 2006; IPCC 2007; Abeysirigunawardena and Walker 2008; BC Ministry of the Environment 2013). However, despite being vulnerable and rapidly changing, these ecosystems are comparatively understudied (Liquete et al. 2013; Savo et al. 2017).

As well as being greatly influenced by climate change, coastal environments are also considered to be some of the most ecologically diverse and productive in the world (Suttles 1990; O’Neel et al. 2015). The Northwest coast of North America is a region dominated by landscape and land-sea ‘edges’ (Turner 2007), due to a complex coastline broken by a plethora of rivers, lagoons, inlets, and fjords, and with a sharp gradient to high coastal mountains (Biogeoclimatic Zones of British Columbia 2013). This diversity in landscapes, combined with the high productivity of North Pacific coastal waters (Fitzhugh and Crowell 1988), correlates with high biological and cultural diversity in the study area (Turner et al. 2003; Loh and Harmon 2005; Berkes and Davidson-Hunt 2006; Turner 2007; Brandt et al. 2014; Thornton 2017).

Indigenous Peoples recognize and mark this diversity through landscape terminology and place names. Northwest Coast Peoples have a rich geographic nomenclature and dense toponomy, which embody nuanced perceptions of the coastal and upland environments, including thousands of features on the landscape (Thornton 1999; Cruikshank 2001; Thornton 2008; Johnson 2010; Johnson and Hunn 2010; Thornton 2012; Anderson 2014). Focusing just on the three languages included in this study region, local peoples have a plethora of terms for weather observations, movements, and patterns (Edwards 2009; Roberts 2009; Lachler 2010; DeVries 2014a, b; Anderson 2018). It is important to relate Traditional Ecological Knowledge about resource, landscape and weather fluctuations to the local languages to understand the landscape history. English terms for places tend to be one dimensional, perhaps naming a location after a famous person with no link to the area named, or simply referring to a single feature in a landscape (Thornton 2008). However, by the virtue of the complexity of Indigenous languages, they can convey not just a word which labels a place, but the geomorphological, cultural, and resource use history through time as related to a location (Thornton 2008). For example, in Glacier Bay, Alaska, the English name simply names a bay with a glacier (‘Glacier Bay’), while the Tlingit name of Sít’ Eeti Geeyi, which means “bay taking the place of the glacier”, is signifying the process of how the bay formed as a glacier receded, and thus describes a geomorphological event (Thornton 2008).

Because Indigenous groups have lived in, and in many cases cultivated (Deur and Turner 2005; Comberti et al. 2015; Thornton 2015; Thornton et al. 2015), their landscapes for millennia, many have recorded a multitude of environmental and biodiversity changes and can correlate these along both long-term and at more immediate timescales, as well as the struggles and exigencies of dealing with the impacts of change. Thus, the results presented in this article represent the observations made by the vast majority of the research participants according to phenomena they encounter year to year in situ and in vivo according to the places they inhabit and the resource hunting, fishing, gathering or other activities they habitually engage in, and show a broad overview of the changes noticed in the region by the participants of this study.

Today, Indigenous Peoples in this region, as elsewhere (Nadasdy 1999; Smith 2002), are insisting that their knowledge systems be considered both independently and alongside of modern science (rather than merely through it) as basic sources for understanding and responding to environmental change and being included as full partners in research (Thornton and Scheer 2012; Cuerrier et al. 2015). The United Nations’ recent establishment of a ‘Local Communities and Indigenous Peoples Platform’ for the purpose of accelerating the inclusion of Indigenous Peoples (IPs) knowledge systems within the UNFCCC process is an important step in this direction (UNFCCC 2017).

Within the Pacific Northwest, this study is focused specifically in the region of Southeast Alaska and Northern British Columbia. This refugia area is diverse geologically, geographically, and ecologically (Clague and Mathewes 1996; Dixon 2001; Brandt et al. 2014; O’Neel et al. 2015), and also culturally, from the ways that people engaged with their resources, all the way through to how they adapted and responded to ecological and geographical change (Langdon 1979; Fedje and Mathewes 2005). While each culture is uniquely distinct, particularly the Haida, their origin stories show interconnectedness (Emmons and de Laguna 1991), which can also be seen in the histories of intermarriages and trading routes between these three groups. Despite the above differences, there are similarities in the species that people utilise across this Pacific Coastal region, which is important when identifying regional keystone indicators, and seeing how people respond to climatic and ecological change broadly. Thus, while the authors recognize the distinctions between each of these cultures, and communities, in this article we draw on broader trends to connect adaptations and species use across the entire region, to chart how peoples’ perceptions are coalescing on ecosystem change, and responses to keystone indicator species, rather than focusing on the perception and response of every community in-depth.

As recent shifts in climate further modify the coastal environment, the ability of Indigenous Peoples to effectively maintain customary uses of these coastal areas is being challenged, as are their previous adaptation strategies and reciprocal relationships with the ecosystem. By looking at the effects of climate change through the multidisciplinary lenses proposed here, and focusing on important cultural and ecological keystone indicator species, we can examine the relationships between Traditional Ecological Knowledge (TEK), Cultural Ecosystem Services (CES; MEA 2005), western science, co-varying diversity values, and impacts of, and current responses to, climate change among Indigenous peoples in this sensitive area.

## Cultural keystones and cultural keystone indicator species

This work suggests the utility of focusing on a portfolio of cultural keystone indicator species to understand and respond to important climatic changes in the northern Pacific Northwest Coast of North America, especially as concerns the biodiversity and provisioning ecosystem services that support Indigenous livelihoods. While not all changes regarding biodiversity can be directly linked to the influence of climate change, climatic shifts constitute a major driver in species distribution, abundance, and health throughout the northern Northwest Coast.

Ecological Keystone Species (EKS; Paine 1969) are species that have a disproportionately large impact on the structure and functioning of the ecosystem relative to their abundance (Power et al. 1996). The idea of an EKS has since been expanded to include ‘Cultural Keystone Species’ (CKS; Garibaldi and Turner 2004, p. 4), described as “culturally salient species that shape in a major way the cultural identity of a people, as reflected in the fundamental roles these species have in diet, materials, medicine, and/or spiritual practices”. Additionally, since EKS are often disproportionately affected by climatic change, and thus can be used as climate indicators (Siddig et al. 2016), EKS that are also CKS and are monitored by local people would be even more useful in recognizing sensitivities and biological reactions to environmental shifts. We propose the term Cultural Keystone Indicator Species (CKIS) to recognize these important plants and animals.

A CKIS may be defined as *critical species of both cultural importance and perceptual salience in relation to environmental change*. While culturally important species have been used as guides in conservation and restoration research (Garibaldi 2009; Charnley and Hummel 2011; Thornton and Kitka 2015), few studies have considered how these key species can be indicators of climatic change (Siddig et al. 2016). We suggest that by examining complexes of climate sensitive, culturally important species, the multiple interactions that exist between species across both seasons and ecotones will become more legible, and a richer understanding of people’s perceptions of climate change will emerge. To demonstrate these intraspecific and climatic links, we draw out several examples of CKIS, and by tracing outward from local and traditional knowledge of these keystone indicator species, we examine data gleaned from ethnoecological sources to document and evaluate how climatic changes are cascading (Turner and Clifton 2009; Wyllie de Echeverria 2013) through biological and cultural systems, and how local communities are responding to these changes. Using CKIS, which emphasizes important species in cultural knowledge and practice, allows us to focus on these causal links and the changing relationships between climate, culture, and ecosystems in a coordinated and policy relevant way.

While a review of the ethnographic literature (cf. Wyllie de Echeverria 2013; Comberti et al. 2015) suggested the pathway towards the CKIS concept, the several plant and animal species that emerged as potential cultural keystone indicator species (CKIS) were informed directly from our interview data and strongly linked to literature on cultural and ecological keystone and indicatory species, and include: salmonberry (*Rubus spectabilis*), blueberries (*Vaccinium* spp.), salmon (*Onchorynchus* spp.), and deer (*Odocoileus hemionus sitkensis*). In this paper, we discuss how each of these chosen groups are both indicator and keystone species, and how they can demonstrate the CKIS concept by linking it briefly with the ethnographic data collected with this research. In some cases, it is more relevant to use a wider classification of ‘functional group’ for a CKIS example, as there may be several related species that hold a similar cultural and ecological role. All five major species of Pacific salmon, for example, are treated as a ‘functional group’ in the salmon CKIS case study, as they have similar ecological requirements and cultural uses, despite differing life cycles. Similarly, several species of blueberries and salmonberry are aggregated into one functional group of ‘berries’ for the purposes of this analysis, as interview data show that these species hold similar ecological and cultural roles in the region, despite variation in patterns of harvest and use.

## Methods

### Setting and community selection

**Fig. 1:**
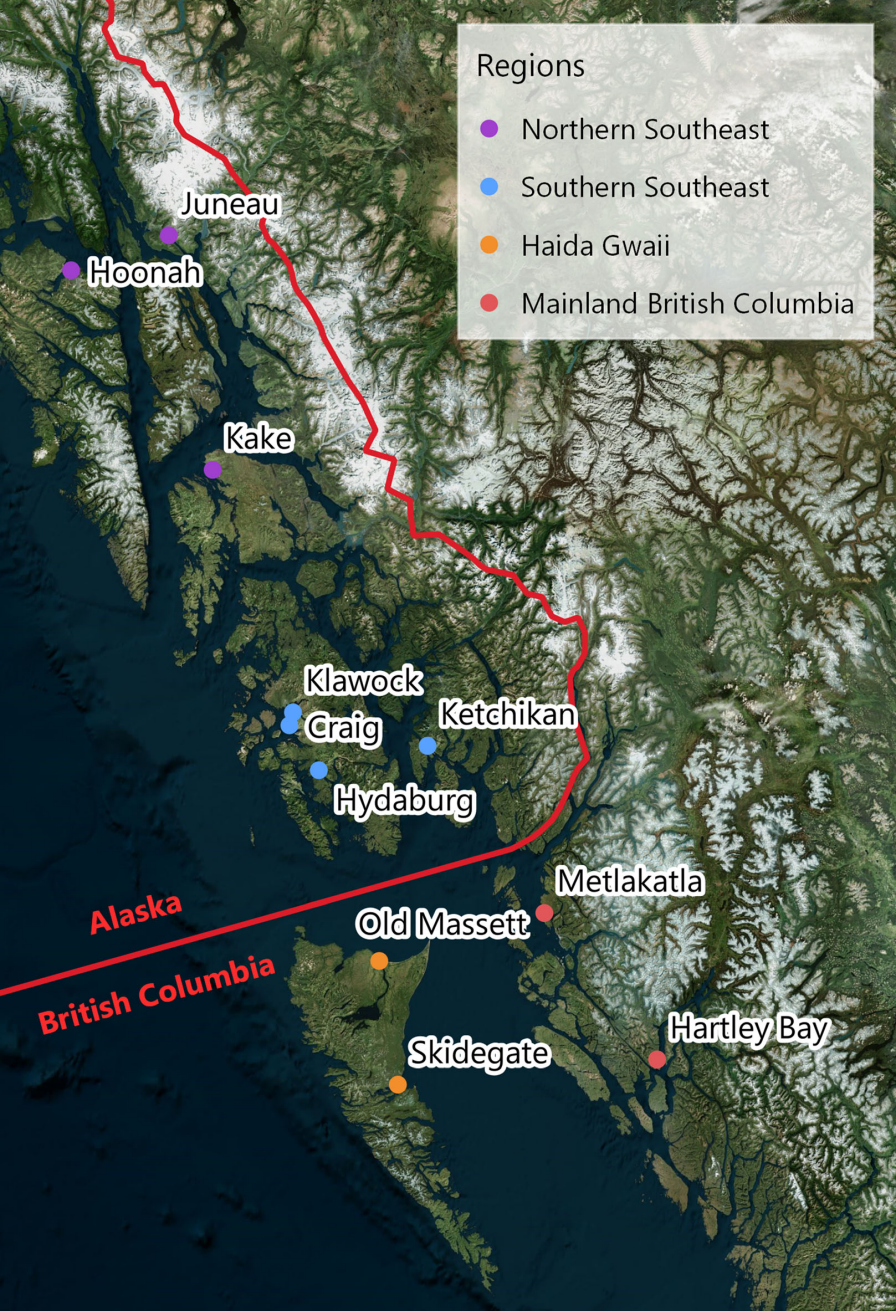
Map of communities visited during this fieldwork on the coast of British Columbia, Canada, and Alaska, United States of America, and showing the four subregions linked to the climatic and geographical zones. Image created by Conrad Zorn, University of Oxford

Research was carried out in northern coastal British Columbia and the panhandle of southeast Alaska in the Cascadia bioregion (Cascadia Institute 2010) between August and October 2014, and in February 2015. While this area is geographically united by coastal ecosystems, it is biogeographically, culturally and geopolitically diverse. Eleven communities were sampled: Juneau, Hoonah, Kake, Klawock, Craig, Hydaburg, and Ketchikan, Alaska, and Metlakatla, Hartley Bay, Skidegate, and Old Massett, British Columbia (Fig. 1). Interviewees consisted of long-term residents either of one of the three main coastal Indigenous Nations, Tlingit, Haida, or Tsimshian, or of European descent. The communities were selected based on their representativeness of climatic zones from the outer southern portion of the biocultural region (Haida Gwaii communities) to its northern “inside passage” and outside coast (Tsimshian and Tlingit). Although all three groups are ‘salmon cultures’, like many Northwest Coast groups, The Haida, Tlingit and Tsimshian represent distinct language groups with their own knowledge systems, social structures, and livelihood adaptations to the microclimates in which they dwell (cf. Langdon 1979).

### Qualitative data collection

Research was begun with contacting the Tribal and First Nation governing bodies. These entities, along with other experts, suggested additional local experts with whom to consult. Interviewees were further recruited via chain referral and snowball sampling from previous interviewees, officials, and experts. Research participants were concentrated into two age groups: One group of interviewees consisted of Elders between the ages of 60–90 who were targeted for their long-term perspectives on climate and resource shifts, and the second group consisted of interviewees between 25 and 59, who were selected because they are not only still active harvesters, but many also often have a long-term perspective as well. These age profiles afforded access to significant historical and contemporary climatic and ecological information.

Overall, 96 people were interviewed, approximately 8–11 people in each community, of which all but five were of Indigenous heritage (but these five had lived in the communities a long time). Three interviewees were governmental personnel (all women), and of community members 40 were men and 53 were women (see electronic supplementary material ESM).

The interview consisted of 16 multi-part questions, through which knowledge was recorded on: weather changes; landscape changes; ecosystem services and interactions; resource use; species biometric patterns; historical adaptation techniques; interactions with governmental bodies; stories of change and adaptation events from ancestors and more recently; and planning and adapting for the future. Interviews primarily took place in the person’s home, or in the elder centre in the community, and permission to go to each community was obtained from the governing body of each council before visiting. Where possible, interviews were supplemented with participant observation experiences, such as walks, drives, and berry collecting expeditions for experiential learning, mostly to look at areas where landscape change had occurred, or to investigate the resource availability (primarily berries). As part of protocol, results are being shared with the communities, with copies of interview recordings, transcripts, and final reports distributed to each community at the conclusion of the research.

## Research questions

Three broad research questions were developed which allowed the authors to draw connections between the diverse groups in this region, see the similarities and differences in human responses to climate change and engagement with the biodiversity of their ecological systems, and to examine the responses to climate by critical species across a regional scale, species which also play a relevance in people’s lives. By gathering a wide data set, this also allowed for further fine-grained analysis, beyond the scope of this current article, but presented in future papers arising from this research. In addition, in the results section tables, a representative quote is used for each observation, but a finer analysis of all the range of quotes provided in the interviews as a whole, and full community comparisons, are not possible to represent in this overview article.

1. Are people noticing changes in the climate, and if so, what weather patterns (as indicative of climate changes) are happening, and are these changes affecting how people access, harvest and prepare resources in their territory?
2. Which culturally important species were observed by people throughout this region to be experiencing change, and what do these changes indicate regarding broader shifts in biodiversity?
3. How can Traditional Ecological Knowledge and climate data be used in conjunction to present a comprehensive picture of local climate change effects and inform adaptive responses?

After transcription, interviews and supplemental data were analysed by coding for themes and organizing statements by observation type, with additional geographical ordering. Ethics protocols as per local and University regulations were followed, including a consent form which each participant was guided through and signed prior to interview. In addition to free prior informed consent, participants were able to determine their level of anonymity, attribution, and how their interview data would be shared.

## Results

### Weather patterns

Almost every interviewee agreed that weather patterns had changed during their lifetimes (Table 1). Most commonly, the heavy snows and cold temperatures that used to occur every year, sometimes yielding accumulations up to the eves of houses, are gone; now there is hardly any snow or frozen water. However, there were differing opinions on the intensity and duration of change, and on how much these changes affected resource distribution, harvesting, and processing. While not everyone could place a timescale on changes in weather patterns, those that did comment on shifting patterns observed that the changes had occurred either in the last 8–20 years, or about 30–40 years ago. Research participants also differed in how they characterized rates of change with some describing a pattern of gradual changes, such that they had hardly noticed it until they compared today’s climate with that of their childhood, while others suggested that changes in weather had accelerated in more recent years. Elders especially felt that in the early period of their lifetimes (50–70 years ago), weather was more predictable and stable, and this was noted throughout the whole study area. Since people scattered in various communities noted the above observations, a geographical pattern was not determined, and thus these differing perceptions may be due to the range of microclimates experienced across the Pacific Northwest region, and more how different places are exposed to different winds and temperatures depending on such local geographic factors as exposure and sheltering patterns, rather than latitude. In this overview article, we have generalized the main observations noted, which unfortunately is not able to provide an in-depth discussion of each community represented, and thus caution must be used as this is such a diverse area, however, there is still value in this overview approach. For example, there were differences between the Alaskan (Northern and Southern Southeast) and British Columbian (Haida Gwaii and Mainland British Columbia) communities with regard to observing sea-level rise vs isostatic rebound.

**Table 1:** Table of basic weather observations

Observation	Changes seen	Impacts	Responses	Example participant perspective
Snow	+ Overall decrease since 40–60 years ago + Some isolated years—major snowfall	+ Less available water for plants, animals, streams, etc. from snow melt + In extreme years, species no longer adapted for cold temperatures	+ Altering harvesting patterns to adjust to yearly fluctuations due to changing weather patterns	‘Before snow come to eves of roof—now barely snow in a year’ (CB, Hoonah)
Sun	+ Fewer sunny days	+ Harder to dry food because of lack of sunny days	+ Drying indoors or with fans, or stoves + Freezing seaweed until there is favourable weather	‘Used to get good sunshine in summertime’ (Mho, Hoonah)
Rain	+ More rain overall + More heavy rain events	+ Washouts and mudslides	+ Harvest in other places, repair areas	‘I’d say…about 30 years ago or so…there’d be a good summer and a bum summer, and it finally went to…now it’s just too much rain’ (AG, Hoonah)
Temperature	+ Air and water getting warmer	+ Changing the behaviours and distributions of animals and plants + Sun is hotter now when out	+ Travelling further to get things + Adapting harvesting patterns + People getting sunburned	‘It used to freeze outside… before we had really freezers, you know, so I know it’s a lot warmer, there’s not as much snow; if there is snow, it [soon] rains and it disappears’ (RDe, Kake)
Storms	+ Becoming more unpredictable and frequent	+ People don’t know when the weather will stay fine to make it safe to stay out harvesting, or even know when they will be able to go harvest at all	+ People delay harvesting, and miss opportunity, or risk going out and getting caught in inclement weather	‘I think there’s usually some overlap, and some set back–might go a couple of weeks thinking ‘alright we’re in the heart of spring,’ and then all of a sudden it gets cold, and some snow falls, and there’s a storm’ (AC, Hydaburg)

Other changes to observed weather patterns that participants commented on included that the timings of the seasons had shifted, and that there was less definition between the seasons. Research participants observing ‘seasonal shifts’ were referring to the fact that weather typically considered autumnal, like major rainstorms, and associated floral and faunal behaviours, were occurring earlier in the year than before. Correlatively, the lengths of each season were seen to be shifting—sometimes shorter temporally then they used to be, sometimes longer. The observed loss of definition between seasons refers to the fact that the boundaries between the four seasons are blurring. For example, AB (Old Massett) commented on the shifting of spring into summer: “it’s not so pronounced now…one kind melts into the other, without…any visible change.” Similarly, EHA (Hoonah) observed that the seasons were becoming less defined, especially with the decline of snow in winter.

“We had a lot more snow, we had our very definite 4 seasons, very defined…and our 4 seasons aren’t even defined the way they used to be, I mean, we even have…in our beadwork, my auntie Jess Grey…[did] the ‘4 season flowers’ [traditional pattern reflecting the distinct seasons]… and it [is] not as defined anymore…like one winter we had nothing but rain, it was just brown all winter; we didn’t get any snow”.

While these observations cannot be easily quantified, research participants made comments about spring weather feeling more ‘winter-like,’ the end of summer being autumnal, and autumn blending in with winter weather patterns. Since the weather patterns of each season affects how plant resources develop and ripen, and when animal species are ready to harvest, it was noted that the timing of harvesting had changed accordingly.

Shifts in global or regional climate (e.g. warmer winters in the whole bioregion) which increase local variation and unpredictability of weather may pose difficult adaptation challenges. For example, uncertainty in accurately predicting both seasonal weather patterns with regard to dangerous weather (an access issue), and the timing and stage of ripening/maturation (a harvesting issue) have led to participants describing having difficulty judging the best and safest time to harvest. Several participants mentioned that they do not always know when to harvest anymore, as the timings are different from a generation ago, so they must expend additional time, energy, and fuel checking the status of the resources for harvest. Coupled with the uncertainty of risky weather, lack of holiday time from the current work economy, and the high costs of fuel, can make harvesting less feasible. AB (Old Massett) commented that he thought people were also more influenced by other peoples’ thoughts about weather conditions, and less reliant on their observational skills and senses than in the past:

“I think it’s just more, we’re, we have radios, we got TVs, we got people, someone’s always screaming oh it’s going to blow 20, 30 miles an hour tomorrow, gusting a 100… and people hear that, before you never heard that… you know, you went by your own instincts, and you’re, uh, you could read the clouds, and you know, the sky… and listen to the birds and animals and see what kind of reaction they got, that’s you know, how they predicted weather before…now we depend on the radio, and news broadcasts, and weather things to find out what the weather is, so, you know, so, I think more, when somebody screams, you know, bad weather, I think everyone’s going to get scared and hunker down and stay there”.

### Landscape changes

Research participants reported numerous changes in landscape composition, structure, and function due to climate and weather shifts (for example, intense or increased rains or higher tidal levels, Table 2). Sea-level change, particularly rising water levels, was noted throughout the entire study area to varying degrees. However, an important change that was noted only in northern areas of the study is isostatic rebound as a result of glacial retreat. This was also connected to coastal areas getting shallower (KG and AD, Hoonah) and tides being perceived as being lower (KG, AG, LKG, Hoonah). These changes to the landscape, which can also be exacerbated by land-use changes (in varying degrees), affected how participants navigate their territories (e.g. shallower water caused by isostatic rebound made it harder to follow known boating routes) or affected the abundance and distribution of resources (e.g. erosion of slopes caused by increased storms).

**Table 2:** Table of observed changes in the landscape

Observation	Changes seen	Impacts	Responses	Example participant perspective
Glaciers and sea ice	+ Less floating ice + Sea glaciers have retreated onto the land	+ Warmer temperatures + Less water available in the spring, glacial melt in streams	+ Adaptation on techniques to moderate water levels in streams	‘Now there ain’t enough ice to make a cocktail, there’s nothing’ (AG, Hoonah)
Isostatic rebound	+ More in Alaska, esp. northern Southeast + Coastal bays getting shallower	+ Boats being damaged, motors hitting rocks + Less floating ice	+ Moving boat moorage sites + Changing travel routes	‘With the ice receding, the land has risen quite a bit’ (AG, Hoonah) ‘You could walk across to Graveyard Island, and when I was a kid they used to anchor seine boats there!’ (AG, Hoonah) ‘There’s some places up the bay where I used to run in and out of, oh I ran through there, it’s shallow here! You know, it’s getting shallower…that’s uplift…and it is climate change, it’s a long one, the glaciers are retreating’ (KG, Hoonah)
Tide Levels	+ Higher tide levels + Increased number of ‘high’ high tides (yearly maximum high tides)	+ Losing land (under water) + Harder to harvest clams, cockles, and other beach resources	+ Moving houses/usage areas	‘Our culture camp, the tides have taken out so much of the gravel there, there’s hardly any grounds left on the camp, you know, it’s being washed out so much…so it seems like the tides are bigger…it’s been washed away a lot, and where our camp used to be, a big area…of the beach, [is] receding” (RDe, Kake)
Erosion	+ Due to heavier rainfall + Decreased plant cover from deforestation + Beach erosion from higher tides	+ Plants and soil washed away + Shoreline changing (in conjunction with deposition, Old Massett)	+ Adjusting uses and access to the area	‘So there’s been erosion, there was two contributing factors to that, they did dig up our sandbar, and pulled a bunch of sand out of there, but also I believe the water is rising slightly’ (AC, Hydaburg)

### Linguistic context of weather and landscape observations

In all three languages/five dialects (Tlingit, Tsimshian, and Alaskan, Old Massett, and Skidegate Haida) encompassed in this study area there are individual words and language phrases (both nouns and verbs) that relate to the weather observations noted by participants in this research, including snow, sun, rain, extreme temperatures (both cold and hot), storms, and tides, the existence of which further illustrate the knowledge of weather patterns across the region (Edwards 2009; Roberts 2009; Lachler 2010; DeVries 2014a, b; Anderson 2018). In addition to a variety of words and phrases referring to these phenomena occurring in general, some specific terms were also used, and these are detailed below. In both Tlingit and Old Massett Haida, there were phrases that related to snow being quite deep, relevant to the heavy snows and cold spells noted, and in Old Massett Haida, there was a phrase for people getting sunstroke from the heat, which could indicate that the temperatures on sunny days could get quite warm, potentially burning people, another observation noted. All five dialects detailed terms for storms, rough waters, wind/rain storms, squalls, gusty, and blowing hard. Tlingit and Tsimshian additionally had terms for snowstorms and these two languages, plus Skidegate Haida, had phrases around being unable to travel due to storms and finding shelter from storms, another noted observation. Surprisingly, erosion was the only landscape change which was not noted in all dialects/languages. There were no terms identified relating to erosion in Alaskan Haida, and a term for ‘landslide’ was the sole term found referring to erosion, in Tsimshian and Skidegate Haida. Phrases around mud, soil, and dirt were found in Tlingit, Old Massett Haida and Skidegate Haida, and Tlingit and Old Massett had specific terms for the ground being softened by rain and turning to mud, Old Massett and Skidegate Haida had terms for washouts, whether by gullies, rivers or wave action, and Tlingit also had terms for snowslides, avalanches and rockslides. Finally, while not every dialect was listed as having a term for red tide, Old Massett and Skidegate Haida did acknowledge its presence with specific phrases, and the particular reference to something being ‘poisonous from red tide’ (Old Massett) shows people’s awareness of the severity of red tide. Terms for weather were also linked to biological resources. On two occasions phrases were used to relate food processing to weather patterns. In Tlingit, Tsimshian, Old Massett Haida, and Skidegate Haida, there were phrases describing foods being dried in the sun, and in Tlingit only there was also mention of fish air-dried in freezing air. Old Massett and Skidegate Haida both link words describing rain with two birds, sandhill crane and black oyster catchers, both said to make a lot of noise when it is going to rain, showing an association between weather and an indicator species.

### Human adaptation to biodiversity changes

Most critically for the natural resource economies of North Pacific coastal rainforest communities, changes in temperature and precipitation have brought changes to ecosystem composition and function, in turn affecting resource abundance, access, and use (Alaska Coastal Rainforest Center 2013). Respondents observed changes ranging from broad-scale landscape composition shifts, to finer scale changes concerning behavioural adaptation, and individual size, abundance, distribution, and quality of resources (Table 3). Non-climatic factors are also affecting resource use, including social and technological change, competition, and scarcity due to other anthropogenic causes (further elaborated in discussion). Despite these changes, important plant and animal resources remain available, although accessing them may require more energy and inputs.

**Table 3:** Table of observed changes in resource use

Observation	Changes seen	Impacts	Responses	Example participant perspective
Shift in species composition/changes in migratory patterns of current species	+ Species shifting or expanding range more common than completely new species + Small migrating birds staying for the winter (robins and blue jays) + Moose coming from mainland	+ New species present + Species moved from where usually are	+ New species to harvest + Hard to find species when they’ve moved	‘Something’s going on…difference in the air, and just last week…the robins are still here…crash into my bay window…I said ‘you rascals,’ you’re supposed to be headed down south, what are you doing here?…and bluejays, and…I find that really, really strange, especially after that big thunderstorm, we had a little flurry of snow, about a month ago…everybody was shocked, and, so there’s things happening in the air that [are] unexplainable…the birds are not leaving’ (HC, Hartley Bay)
New species appearing	+ Mostly linked to extreme weather events, such as ‘unseasonably’ cold or warm years + Tropical fish	+ Can interfere with usual species + New resources to hunt/fish for	+ Changes species harvested	‘So we are starting to see more fish from the south, from California, and we never did before’ (AB, Old Massett)
Species disappearing	+ More species declined than completely disappeared	+ Less available for harvesting	+ Limit harvesting	‘Our birds are less and less…you don’t see them in the flocks that we used to see them’ (WC, Hoonah)
Changes in behaviour	+ Whales coming closer to shore, behaviour they exhibit when it’s stormy + Salmon swimming deeper (because of warmer water temperatures, to get to cold water) + Declines in salmon fecundity due to lower water levels (Scannell 1992) + Deer moving further away from human development and up mountains (less inhibited with lower snowfall), harder to hunt	+ Fewer salmon to harvest, shared and put up for winter + Harder to hunt deer	+ Changed resource use and sharing + Changed ceremonies—not able to use deer, salmon or other traditional meats in potlatches	‘We’re watching the whales down there, and the whales…for us, they are [a] weather…sign…and that would be blackfish or killer whale…so when they are going past the village, the old people will say they are headed out to the ocean, they are going to feed on the ocean…and they call the Pacific the big sea…in our language, and it’s calm and now they are able to go out and gather, they got all the room in the world, and so then when they come in and go past the village, that means the Pacific is acting up…and they are coming into feed…close to shore, because there’s a storm coming, and so they’re coming in’ (HC, Hartley Bay)
Diseases	+ Higher levels of red tide due to warm water + Higher rates of worms and flies in deer and fish when preserving by drying and smoking, and in harvested berries, due to warmer autumn temperatures + New varieties of pests around plant resources	+ Meat and berries of lower quality	+ People can harvest/use less	‘The sockeye, when, the warmer the water the more worms they get in their stomach’ (AB, Old Massett)
Size of plant or animal	+ Fish getting smaller (also linked to overfishing and pollution, not just climate change)	+ Same limits, so overall less food	+ People don’t have as much fish to eat	‘And they are getting smaller… one year they had 31 lb [14.1 kg], and then 29 lb [13.2 kg], last year it was 27 lb [12.2 kg] won it, this year it’s 26 [11.8 kg]’ (MB, Juneau)
Quantity of resource	+ Flowers blooming earlier, either vulnerability to frosts damaging flowers, or less pollination occurring, meaning less fruit + Deer are moving higher into the mountains due to less snow, and away from development + Salmon are moving deeper in the water, under the nets	+ Fewer berries, deer and salmon + More difficult to harvest	+ Don’t go out to pick berries + Changed ceremonies—using store-bought berries in potlatches instead + Drive into mountains, or hunt from boats, wait for a snow to drive deer down in elevation + Fishing closer to the river	‘Well, right now, it’s kind of extreme, like, right now, we see [salmonberry] blossoms out and we’re in January…and, that means we won’t have berries again…because there’s no bees around’ (RD, Old Massett) ‘Depends on the weather, eh? If there’s not much snow, then all the deers’ up in the mountains… not too many people want to climb…and if there’s snow we can get it on the beach, just travel on a boat and get our deer’ (WB, Kake) ‘We’ve been having a hard time catching a lot of fish sometimes, because…they are staying deeper then they normally, and they aren’t showing and jumping and behaving like they usually do, because the fresh water is so warm due to the lack of snow pack up there, melting’ (AC, Hydaburg)
Quality of resource	+ Too much rain = fruit rot before ripening, or swollen with water and not fully ripening (not maximum sugar potential, no taste) + Not enough rain (or not at the right times) = berries shrivel up and desiccate + With lack of food, deer are leaner, and have a lower quality meat	+ Fewer berries + People don’t want to harvest poor quality meat	+ Don’t go out to pick berries + Changed ceremonies—using store-bought berries in potlatches instead + Less deer eaten	‘It did rain a lot this year, so I think that’s made a difference in our berries, it’s rotted a lot of them…mildew got to them’ (WC, Hoonah) Women like fatty meat, better for salting (HC, Hartley Bay)

Most of the impacts concerning animals emphasize changes in behaviour, abundance and movement, while for plants shifts in quality and quantity of fruits, and timing of flowering and fruiting, were linked to changing weather patterns. Plant distribution was more closely tied to land-use changes (logging, land development, and conservation areas) rather than specifically climate change.

### Adaptation responses

While research participants recognize that significant weather changes are occurring, such changes are not necessarily regarded as unprecedented. Many Northwest Coast communities have experienced major environmental changes, such as glacial movements, tsunamis, and drastic sea-level rise in the past, yet still survived and recovered, resiliently. Despite being wary about the future, most respondents possess a positive outlook regarding their ability to cope and adapt. They view themselves and their culture as always having adapted to environmental change, and thus continuing to adapt to future change. Emblematic of this adaptive capacity and resilience are the many stories of the Flood in Northwest Coast oral history, in which clans or communities survived inundation from massive sea-level rise by seeking refuge on the tops of high (2000 + foot) mountains, and then re-establishing themselves on the altered land in the aftermath (de Laguna 1960, 1972; Emmons and de Laguna 1991; Hunt et al. 2016). These and other “epitomizing events” (Fogelson 1989), markers of peoples’ resilient and adaptive histories and identities, were often referenced in our interviews. Now research participants focus on how they are adapting to changing resource accessibility, availability, harvesting and processing techniques, knowledge systems, and co-management arrangements, in addition to broader climate changes (Table 4). In addition, they discussed the intricacies of passing down knowledge to future generations, which is not included in the table, but further evaluated in the discussion. Research participants also described changes to the intensity of resource use in their responses. Many respondents detailed that members of their community do not gather as much as they used to, particularly the younger generation, and that, in addition to access issues tied to weather and sea-level changes, access to resources was further limited by working hours (jobs), permit applications, harvesting regulations, and costs of fuel and boat maintenance.

**Table 4:** Table of observations of adaptation and future resilience

Observation	Changes seen	Impacts	Responses	Example participant perspective
Harvesting	+ Different weather changes patterns and timing of resources + Stormier—harder to get out when things are ripe	+ Harder to harvest	+ People have less resources	‘It’s hard to get out sometimes, you know, I know when I was expecting it to be there…and when I went looking for it, it was already gone…plus gas, you know, we gotta go check on it’ (MY, Craig)
Processing	+ Drying food harder because of lack of sunny days + Freezers changed timing of when could preserve food—reduced need for immediate preservation, wait for good weather + Drying salmon inside using heaters and fans	+ Harder to process	+ Novel ways to process + Using freezers more	‘I’ve talked to many people, and they say it just can’t be done, but I know it can be done…I, worked with it now until I got it down to a fine art…there’s a temperature gauge on the outside of my smokehouse with a sensor that runs…on the same elevation as my screens…I got screens I put the halibut on…I got to get the temperature up to…120° [48.9 °C] I start timing it, and it take[s] 25 min, and at the end of 25 min I open my door, I put a fan on, and I cut the heat down on…and so I draw the temperature down, and preferably, ah, a 100° [37.7 °C], and not lower than 80 [26.7 °C]…but try to keep it 90–100° [32.2–37.7 °C] in there, and just stay that way until it’s done’ (AG, Hoonah)
Co-management	+ Some positive Alaskan Government and Hoonah Tribe –to authorize renewal of bird egg harvest in Glacier National Park according changing environmental constraints + Co-management of salmon between Alaska and Hydaburg tribal governments	+ Old resources being re-opened + Local knowledge taken into consideration in management	+ Some people excited about these changes	‘I run the management program for the sockeye fishery that we have for subsistence food fish here…and um…we’ve had some real good years, and some bad years, our community, uh, gets grants to help facilitate managing a weir, and uh, getting a fish count and tallying our subsistence take, and so we’ve been basically co-managing that fishery, uh, and we noticed that over the course of the years of that program, the trends in when the populations, uh, when [the] genetically different populations came into the rivers, so that there were distinct populations that had, uh, you know, they didn’t cross breed with each other, they come in June, they come in July, and they come in late August and September, so there are three distinct populations…and that was all sockeye, in a lake system…and so we had, we started to manage them, as three separate stocks, rather than one stock…cause we could overfish the early run, and basically take out the early run…and that’s what had happened, we had depleted the early run down through several years of fishing, being excited that that’s when they come in, and fished them down to nothing, and so our information gave us the tools to, uh, take the pressure off that stock, to ask community members to back off on fishing in June, and, uh, without having to go to the regulatory body and say hey, change this…we’ve already been regulated enough as natives, we don’t need to go to that…self-regulation’s a lot easier…because then you’re taking ownership and you’re changing behaviour, and that’s going to uh, impact and ensure longevity and sustainability of resources in your fishery’ (AC, Hydaburg)
People part of climate change	+ Lifestyles in remote communities very energy intensive and costly “Social Climate Change”	+ Costs of harvesting (e.g. fuel) becoming prohibitive	+ People looking at ways to alleviate energy stress	‘The thing that’s changing is the social change… our people used to depend on gathering, processing fish, smoking fish, smoking deer meat, seal meat and that, but now it’s not [the same]’ (KG, Hoonah)

### Humans as a component of the ecosystem

Another common thread throughout interviews was that research participants recognized themselves as a component of climate change. Current lifestyles in remote communities are heavily dependent on high CO2-emitting fossil fuels. Several hundred years ago, Indigenous Peoples interacted with their landscapes in a more self-sufficient way. In contrast, now significant amounts of high carbon fuels are consumed even in remote communities for livelihood activities, food and other imports, transport, storage, heating, lighting, and so on. Power is typically provided by diesel-powered electrical stations. As a result, people in these remote areas also are contributing to greenhouse gas emissions, which contribute to climate change in relatively small (compared to urban areas) but often increasing amounts (Powell 2015).

KG (Hoonah) emphasized the idea of local people both being affected by and affecting “Social Climate Change”. He states that it is not just the climate that is changing, or the government, or pollution, or the ability to afford fuel, etc., but rather their entire way of life, and how they approach the landscape—“the thing that’s changing is the social change…Our people used to depend on gathering, processing fish, smoking fish, smoking deer meat, seal meat, and that, but now it’s not [the same]”. This sentiment, that climate change is not the only or even the most important impact on local peoples’ present lives, but rather one driver in a concatenation of forces reshaping Indigenous lives, is widely echoed in the literature on climate change and Indigenous Peoples (see, e.g. Crate and Nuttall 2009).

### Case studies

In each of the following case studies, we present why each of the CKIS suggested above qualify as both an indicator species/functional group, and a keystone species/functional group. Additionally, we examine the broad changes that were commented upon in the interviews drawn on for this article and summarize why each of these case studies fits into being a climate indicator, and a CKIS.

#### Pacific Salmon (Onchorynchus *spp.*)

In this CKIS category, we include the five species of salmon on the Pacific Coast: *O. gorbuscha* (Pink/Humpys), *O. keta* (Chum/Dog), *O. kisutch* (Coho/Silver), *O. nerka* (Sockeye/Red) and *O. tshawytscha* (Chinook/King/Spring).

Indicator species: Salmon migrate long distances and utilise a wide range of habitats at different parts of their lifecycle, from the open ocean up to small tributary streams. Thus, they can be used to monitor both fresh water and oceanic conditions, along with stream, riparian, watershed, and upland conditions (Bryant et al. 2008). Overall, all five species of salmon are used to indicate environmental characteristics such as changes in human and natural disturbances in watersheds (e.g. landslides and logging), the condition of the watershed (debris, sediment), stream flow, temperature (both marine and stream), salinity, and ocean currents (Hyatt and Godbout 2000; Gilkeson et al. 2006; Bryant et al. 2008; MOE 2016). While all species fulfil a similar ecological role, some species of salmon are thought to be better indicators of certain environmental conditions. For example, coho is ideal for monitoring the effects of human and natural disturbance on watersheds due to its longer life history in fresh water (Bryant et al. 2008), sockeye prefer colder water, so are ideal for monitoring temperature changes (MOE 2016), and pink salmon are known for their steady population fluctuations, and thus significant changes in population can be tracked easily (Estes 2014). Hyatt and Godbout (2000) detail why Pacific salmon should be considered indicator species, which includes: they are wide-ranging in their distribution, and occur in both marine and freshwater systems; they contain variety with regard to genetics and life histories both within and between species; they are extremely sensitive to environmental cues; there are many long-term studies of salmon populations; and finally, they are extremely relevant both socially and economically to both Indigenous and non-Indigenous people in this area.

Keystone species: Pacific salmon are considered to be an EKS because they are a very important food source for a host of other animals, ranging from large predators, such as bears and wolves, birds (including eagles), predatory fish, aquatic and riparian scavengers, and insects (Willson and Halupka 1995; Cederholm et al. 2000; Hyatt and Godbout 2000; Reimchen et al. 2003), in addition to humans. They are preyed upon at every stage of their life history, from eggs to carcasses (Willson and Halupka 1995). At least 138 species have some kind of relationship with salmon throughout their lifecycle (Cederholm et al. 2000). In addition to being eaten, their carcasses are often moved into terrestrial environments and broken down by detritivores to provide nutrients to the plants and enrich the soil, which provides a pathway for nutrients to connect the ocean and forest (Cederholm et al. 1999; Reimchen et al. 2003; Hocking and Reynolds 2012). The nutrient input of salmon into the coastal environment has influenced the structure and function in this ecosystem, and the yearly contribution affects entire ecosystem survival and reproductive capacity (Willson and Halupka 1995; Cederholm et al. 1999).

Changes observed from the Case Study Data: Participants in this research stated that salmon were a valuable resource to them and discussed that they had noticed changes in their timing, individual population size, population stability and distribution, and behaviour, much of which they link to the climate and weather changes that participants had noticed. In particular, fishers reported that the abiotic feature of warmer water was affecting salmon greatly, causing them to swim deeper in the nearshore, which made them harder to catch, but also causing more worms in the meat (noted in sockeyes), and scale loss (noted especially in cohos). As well, fishers have noted that there has been a general decline in salmon returns and the amount of fish they have harvested in recent years, which they attribute to the changed distribution patterns causing fish to alter where and when they are migrating (swimming deeper), and the fishers’ access to the fishing grounds (regulations/adapting to season opening times and high cost).

Collectively, these observations provide important commentaries on the social-ecological impacts of climate change. They are also emblematic of the utility of focusing on CKIS. Because the responses of salmon to different abiotic features can be measured both scientifically and through human observations in the context of livelihood practices, so too can oceanic and terrestrial watershed and riparian conditions be tracked through the salmon populations and their behaviour, providing sound indicators of climatic changes.

#### Deer (*Odocoileus hermionus sitkensis*)

Indicator species: Sitka black-tailed deer prefer to live in a mixed habitat throughout the year. Their specific use of different habitats and their general presence and movements between landscapes throughout the year, indicate the quality of old growth forests, open younger forests, and forest edges, and floral diversity, as deer have a varied herbivorous diet of herbaceous and woody plants, characterized by consistent features, such as digestibility (Hanley 1996; Lee and Rudd 2003; Schoen and Kirchhoff 2007). Biologists (cf. Hanley 1996) consider deer an indicator species on the basis of the following characteristics: they have large home ranges which they migrate throughout in a seasonal pattern; they make use of different habitat types, seasonally, including varied food sources and canopy cover options; and they are valued by local people, primarily as a food source, which makes them ‘socially relevant’ (Hanley 1996). Deer particularly indicate when undisturbed habitat is lost (Lee and Rudd 2003), as this relates to their forage and movement requirements.

Keystone species: Sitka black-tailed deer are considered a keystone species because their presence has great effect on the landscape. When they are removed from the system, the floral architecture and diversity, the processing of minerals such as nitrogen, and the local soil make-up (Cobb 2014) will often change. Their availability also affects populations of megafauna (wolves, black bear and brown bear), including humans, who rely on them as prey (Schoen and Kirchhoff 2007).

Changes observed from the Case Study Data: Participants in this research found that Sitka black-tailed deer greatly tailor their seasonal migration and habitat use to reflect changing weather patterns. The abiotic factor most closely tied to their distribution is snow. Because air temperatures overall are warming, smaller amounts of snow cover may adhere and endure, thus facilitating migrations higher into the hills and mountains. These migrations, in turn, may render deer less accessible to hunters. Alternatively, if there is a deep and heavy snowfall, as is evident with increased storms and severe weather events in the region due to the changing climate, deer will be driven closer to a human presence. The snow levels will also impact available browse, which will affect how the deer population shrinks and expands. In addition to snow levels, warming temperatures also affect the quality of the meat, such as when they put on their winter fat, and higher incidences of disease, such as ticks. Since the effect of these abiotic factors on the variation in population levels is not well understood, this is leading to issues of asynchrony between governmental regulation on hunting times, and when deer are actually available, which impacts the local people’s ability to harvest this traditional resource.

From these observations, it can be seen that deer can be used to indicate snow levels and distribution, both in the current year and following years, based on their location and population size in response to this abiotic factor. As deer are heavily impacted by quantities of snow fall, where they distribute themselves can indicate current snow depth levels. If deer are low in elevation, it typically indicates snow on the mountains, while if deer are absent from lower elevations, it indicates the availability of browse (and lack of snow) at higher altitudes. In this way deer can be tracked throughout the year to monitor snow. It is worth noting that in this study area, the evidence for the inclusion of deer as a culturally important species, and thus an example of CKIS, is not as strong in Old Massett and Skidegate, Haida Gwaii, as it is in the other communities in this research area. The main reasons why the evidence for this is weaker is because the deer are an introduced species on Haida Gwaii (Gillingham 2004), and are considered pests by many people, particularly due to their devastation of the local flora through over browsing (which was frequently mentioned in interviews from this study). Despite being an invasive species, however, some people have come to rely on deer as an important source of meat, and in interviews noted significant behavioural, phenological and population observations. As May Russ (Old Massett) commented “but, you know…on one hand people want to get rid of them, because they’re not indigenous to the island, and then there’s, on the other hand, there are people, it’s becoming a food source for them”. Thus, over time, deer may continue to rise to full CKIS status in Haida Gwaii and become highly valued, although it is not currently as strong a CKIS example as it is in the communities in Alaska and mainland British Columbia.

#### Blueberries (Vaccinium *spp.*) and Salmonberry (Rubus spectabilis)

In this CKIS category, we include Salmonberry (*Rubus spectabilis*) and the blueberry species (*Vaccinium* spp.) that commonly occur in the study region: *V. alaskense* (Alaska blueberry), *V. ovalifolium* (ovalleaf blueberry), *V. caespitosum* (dwarf bilberry) and *V. uliginosum* (bog blueberry).

Indicator species: Plants are commonly used to indicate certain soil/moisture characteristics (Halverson et al. 1986; Klinka et al. 1989). Salmonberry and blueberry have slightly different ecological requirements, and thus their presence is used to indicate different soil regimes. Salmonberry require locations that have wet and moist conditions and are rich in nitrogen (Halverson et al. 1986; Klinka et al. 1989). Blueberries require habitats that are acidic, poor in nitrogen and wet, such as bogs (Klinka et al. 1989; Hilty 2015; USDA Forest Service 2018b). Because salmonberries like a moist environment, their presence often indicates a close source of water (Halverson et al. 1986). Blueberries hold two very different ecological roles. They frequently appear shortly after a disturbance occurs, such as clear-cutting, and there is a more open ecosystem, as they can take hold easily with more light and a lack of competition and can thus indicate land-use changes. However, they are also a common species in old growth ecosystems (due the open forest structure), thus indicating the presence of mature forest (USDA Forest Service 2018b). These two groups of plants are ideal indicators because they are closely monitored by harvesters, common enough to be seen in many places, and are affected by changing weather patterns.

Keystone species: Both salmonberry and blueberries are keystone species for the role they play as forage and habitat for various animals, including humans. Salmonberry provide cover and nesting sites for many local birds and mammals, such as red squirrels, mice, black bears and beavers (USDA Forest Service 2018a). As well, many parts of the plant are eaten and are a vital food for a large number of animals. In Cascadia, leaves and twigs are an important food source for local ungulates such as deer, mountain goats, elk, and moose, as well as smaller mammals (i.e. rabbits, porcupine, beaver) and fruits are eaten by a wide range of local species from birds (i.e. grouse, songbirds, American robins) to small and large mammals (i.e. squirrels, foxes, mice and rodents (primarily the seeds), and black and brown bears) (USDA-NRCS 2012; USDA Forest Service 2018a). Finally, the nectar is an important food source for bees, butterflies, other insects, and hummingbirds (USDA-NRCS 2012; USDA Forest Service 2018a). In addition to their food value, a salmonberries’ rapid growth and dense belowground root and stem systems bind soil well, making them important species for stabilizing eroded or disturbed sites (British Columbia Nature 2002; USDANRCS 2012; USDA Forest Service 2018a).

Blueberries play a very similar role to salmonberries. They are thought to provide cover to mainly bigger animals due to the height of the plants (USDA Forest Service 2018b), and in terms of food availability, leaves, flowers, and fruits are utilized. In this region, leaves are an important food source for both local ungulates such as deer, mountain goats, and elk (USDA Forest Service 2018b, c) and for the larval stages of Lepidoptera, several species of which feed solely on *Vaccinium spp*. (particularly *V. uliginosum* from this study area, Natural History Museum 2019). It is particularly a favourite food of black-tailed deer (*Odocoileus hermionus*) in Western Washington (USDA Forest Service 2018b). As well, fruit are eaten by a wide range of local species from birds (i.e. grouse, ptarmigan, pheasants, songbirds) to small and large mammals (i.e. squirrels, foxes, and black and brown bears) (USDA Forest Service 2018b, c). Finally, the nectar has been documented as an important food source for several species of both long and short tongued bees (Hilty 2015). In addition to its food value to animals and humans, blueberries are very economically relevant to local people, both commercially and with wild harvesting (https://www.adfg.alaska.gov/sb/CSIS/). Blueberries can grow from seed, cuttings, or damaged parent plants quickly, and thus can spread (or be planted) to cover cleared areas rapidly, providing stabilization and a food source in disturbed sites (USDA Forest Service 2018b, c).

Changes observed from the Case Study Data: Participants interviewed reported that both salmonberries and blueberries were very important to them for food and, particularly with regard to blueberry, an economically viable wild harvest, especially noted in Hoonah and Kake. Both berries are significantly influenced by changes in weather at critical growth stages. First, Indigenous observers suggest that earlier and warmer springs are causing plants to bud earlier than normal. This pattern of earlier flowering often leads to a disconnection with pollinators which either have not arrived (e.g. hummingbirds), or have not become active (e.g. bees), in turn decreasing the amount of fruit forming. Further damaging to fruit formation is the increasingly common pattern of a warm start to the spring that encourages early bud growth that is subsequently killed by a later frost. Even if fruits do set under these variable conditions, the quality and quantity can vary greatly depending on the summer and autumn weather conditions. If the conditions are too wet, the berries either become saturated with water and don’t produce the necessary sugars to fully ripen, or they rot before ripening. In addition, increased rainfall can cause immature forms of insects and worms (species not specifically identified by the interviewees) to reproduce more frequently in the berries, which renders them less favourable for consumption. Alternatively if the conditions are too dry, as evident with hotter, drier weather spells in early summer, the berries will desiccate and dry out before they ripen.

From these observations, it can be seen that changing late winter, spring, and summer weather conditions can be tracked by the quality of these CKIS fruits. Furthermore, observing the phenology of salmonberries and blueberries can indicate critical changes in climatic conditions during the growing season.

To link the above three CKIS examples to the linguistic literature, in Table 5 we illustrate the Indigenous names for all the CKIS examples in this article. Additionally, the language references (Edwards 2009; Roberts 2009; Lachler 2010; Anderson 2018; DeVries 2014a; b) were searched for additional terms relating to the CKIS examples. All three languages had various terms relating to salmon in general. These included terms around seasons and locations of fishing, spawning, fishing tools, life stages, anatomy, processing, and mythology. There were also terms that related specifically to each of the five species. The three Haida dialects had the most number of terms: solely naming 10 terms specific to coho, 15 terms specific to chum, and six terms specific to sockeye. Both Tsimshian and Haida had a total of 6 specific terms for pink, and all three languages, Tlingit, Tsimshian and Haida, had a total of eight specific terms for king. Deer had terms in all three languages that related to hunting and movement, life stages, anatomy, processing and preparing skin and meat, and diseases. While generic terms for berries were found in Tlingit and Haida, relating to flowering, size and stage of ripeness of berry, and food use, specific terms for blueberries were only found in the three Haida dialects, covering anatomy, location, and the action of gathering, whereas specific terms for salmonberries were found in all three languages, relating to season and location, anatomy, colour and size of berry, medicinal usage, and mythology.

**Table 5:** CKIS names from the three languages throughout study area

English name	Scientific name	Tlingit name	Tsimshian name	Alaskan Haida name	Old Masset Haida name	Skidegate Haida name
Salmon, chum	*O. keta*	téel’^a^	g̱ayniis^c^	sk’ag^e^	sk’ag^f^	sk’aagii^g^
Salmon, coho	*O. kisutch*	l’ook^h^	üüx^c^; ẅa̱a̱x	táay^e^	taayi^f^	táay.yii^g^; táay.yiigaay^g^
Salmon, king	*O. tshawytscha*	t’á^a^	yee^c^	táa’un^e^	taawan^f^	taaGun^g^
Salmon, pink	*O. gorbushcha*	cháas’^a^	sti’moon^c^; stmoon^c^	ts’at’áan^e^	ts’at’aan^f^	ts’iit’an^g^; ts’iit’aan^g^
Salmon, red	*O. nerka*	Gaat^a^	misoo^c^; müsoo^c^	sGwáagaan^e^	sGwáagaan^f^	taaxid^g^
Deer	*O. hermionus sitkensis*	Guwakaan^a^	wan^c^; wa̱n^c^	k’áad^e^	k’aad^f^	k’aad^g^
Blueberry (generic and oval-leaved)	*V. ovalifolium*^j^	kanat’á^a^; kakatlaax^b^	smmaay^c,d^; smmay^c^	Hldáan^e^; hldáan Gadg^e^	hldaan^f^	hldaan^g^
Blueberry, Alaskan	*V. alaskaense*^j^	naanyaa kanat’aayí^a^; naanaa kanat’aayí^b^	–	hldáan kidg^e^	–	hldaan^g,h^
Blueberry, bog	*V. uliginosum*	ts’éekáxk’w^a^; láx’loowú^b^ (swamp); ts’éekáxk’w^b^ (mountain)	–	Gáan xáwlaa^e,i^; hlGu Gáanaa^e^	HlGu Gáanaa^f^; Gáan xáw’laa^f,i^	tllga GaanGa^g^; Gaanxaaw’laa^g^
Blueberry, dwarf	*V. caespitosum*	kakatlaax^a^	mihaał^c,d^	–	–	–
Salmon berry	*R. spectabilis*	Was’x’aan tléigu^a^; Ch’a aanáx tleikw^b^	makooxs^d^; ma̱g̱ooxs^c^; ma̱ḵ’oxs^c^; ma̱ḵ’ooxs^c^	sk’áwaan^e^	–	sk’aawGan^g^

^a^Thornton 2008; ^b^Newton and Moss 2009; ^c^Anderson 2018; ^d^Thompson 2003; ^e^Lachler 2010; ^f^DeVries 2014b; ^g^DeVries 2014a; ^h^*Vaccinium alaskaense Howell* (seems to be ovalifolium); ^i^Also a name for saskatoon berry; Note: hldaan radaga; hldáan radga, hldáan radg = berry of *Vaccinium ovalifolium* (S, M H); hldaan qidga; hldáan qidga, hldáan qidg = berry of *Vaccinnium alaskaense*

## Discussion

This study suggests that many changes to biodiversity (some independent of climatic changes) have been documented by local Indigenous experts as part of their livelihood engagements on the land and sea. These changes to biodiversity include several examples of species completely disappearing (e.g. violets, orchids, migrating ducks and geese, bees, horseflies, frogs, toads) from local environments, as well as new species appearing to become established (e.g. giant squid, tropical fish, starlings, tall invasive grass, mackerel, misc. insects and small invasive herbaceous plants). Dragonflies were an interesting case, as one participant reported that they were decreasing (Old Massett), while another reported that they were increasing (Hoonah). However, because these observations were in diverse locations, specifically northern Southeast Alaska, and Haida Gwaii, this could be an observation that differs regionally, similar to the isostatic rebound versus sea-level rise observations. Other species discussed had experienced range extensions or decreases, or simply variation along the NW Coast (e.g. moose moving onto islands from mainland, bear, elk, yellow cedar). This has led the participants in this research to change what they harvest, such as shifting from moose to deer in some areas, or pursuing newer arrivals, such as tuna. While some of these biodiversity changes are linked to climatic changes, this is a complex ecosystem with many factors at play which influence biodiversity.

In every community, residents observed that animals are not following their usual behaviours. While this is a broad statement, it reflects the regional focus of this article, showing that despite each community being distinct, there were common trends in the observations made by people throughout the entire study area, based on their observations of the natural environment around them. In many cases, this means locals are having to travel farther to harvest, and this travelling can be dangerous with the more unpredictable weather conditions arising from climate changes. Weather patterns, animal behaviour, and welfare are tightly intertwined. For example, in the case of salmon, a reduction in the amount of snow leads to reduced snow pack and less runoff in the spring. This, in turn, may lower stream levels such that salmon lack enough water to reach their upstream spawning areas. The smaller volumes of stream water also tend to be warmer, due to higher air temperatures and a lack of cold-water input from snow melt, further inhibiting salmon viability. While increased rain fall might be thought to counteract the lack of snow, it flows down the watershed at the wrong time of year and may create other problems for salmon spawning and rearing habitat, such as erosion, blockages, and flooding. Warmer water temperatures–in fact any large variations of temperature range–have been shown to have impacts on spawning, incubation, and fry survival and movement (Scannell 1992), which in turn affects the number of adult fish that are available for harvesting in subsequent years. Warmer water also affects fish behaviour and disease vectors. Many of the fishermen said it was harder for them to catch fish as they come into the nearshore now, because when the fish swim towards the rivers to spawn, they are swimming in the deeper, colder water to escape the upper layers of warm coastal water, and thus often swim below the depth of deployed nets, hence reducing catches. Alternatively, some participants mentioned that they are now having to fish closer to the mouths of the rivers, as fish are forced to swim higher up in the water column due to decreasing depth.

This is one of numerous examples demonstrating how local observations of a cultural keystone indicator animal species can reveal how weather conditions and peoples’ interactions with the ecosystems are interconnected and changing with climatic shifts. Another example, this one of a plant species’ response to changing weather conditions, shows clear impacts on peoples’ usage, including changes to the quality, quantity, and distribution of berry species throughout the gathering season. Berries were either described as being either swollen with extra water, or small and desiccated, from differing amounts of rain, and both scenarios affected the taste and quality, and thus peoples’ interest in harvesting and eating them. Participants describe having to replace wild berries at feasts with store-bought berries, as it was more reliable and easier: “I hate to even say that, I have seen times where some… have gone to Costco and bought the blueberries for the parties… [now] it’s a lot easier to just go buy them from Costco” (MJo, Juneau). Quantities of resources also were reported as varying according to climatic conditions, with seasons with extreme weather in either direction resulting to reduced availability for harvest.

There were mixed feelings by interviewees about how knowledge will continue to pass down through generations given the rapid social and ecological changes. There were several communities that had programmes in the local schools, or summer culture camps that provided the younger generations the opportunity to learn and practice their traditions, and make connections between harvesting and traditional activities, with the guidance of Elders. But not all of the younger generation is interested in taking part in this, and it is not clear how older traditional knowledge and new climate change would be reconciled in such forums. However, it is likely that there will be a merging of technology and knowledge, partially to aid in rapid knowledge transfer to counteract loss, similar to that which is being done in participatory mapping studies in the arctic (Bennett 2012; Gill 2013). The younger generations are often proficient at technology and letting them engage with their Elders through the medium of pictures, videos, recordings, Internet media, and interactive maps will facilitate knowledge transfer in new ways.

And while research participants expressed uncertainty about where the future was heading and whether they would be able to continue their traditions, overall we heard numerous research participants comment that they have adapted to major, landscape forming changes in the past, such as the floods and glaciers discussed earlier, and felt hopeful that they would continue to adapt into the future. Participants also described how they were actively adapting harvesting and processing techniques to account for weather changes and uncertainty—for example freezing or jarring resources and using various types and sizes of dehydrators for drying (specifically) fish, seaweed or berries. One person even mentioned that future generations may have to rely on natural resources, and all their knowledge will be useful to allow their communities to adapt to these changes.

Throughout these interviews, and also in previous research, it can be seen that climate change impacts are not the only thing to affect Indigenous Peoples in coastal ecosystems; it’s just one of many challenges they are facing. Sometimes climate change was difficult to tease out of all the other changes. The other changes that people in this region deal with, and which were mentioned in the interviews, are: commercial and sports fishing, overfishing in general, logging and deforestation, pollution (sewer outfalls, boats), and rules regarding resources that are made with little collaboration between communities and governmental bodies. In several of these cases, changes that appear to be attributed to climate change may in fact be caused by something else, several examples of which are detailed below.

Overfishing was a major factor in the lack of available resources close to villages. Crabs, shrimp and fish are being decimated nearshore due to increased levels of fishing, particularly as seen in charter and sports ventures (LKG, Hoonah). Also, when looking at fishing derby records, participants commented that the winning fish was getting smaller (CB, Hoonah). On top of warming waters, changing behaviours, and fecundity, overfishing is only exacerbating the issues.

Alder trees (*Alnus* spp.), an early colonizing species, can invade and shade out bushes through vegetative succession, reducing berry harvest. However, MJo (Juneau) explained that clearing the edges of the roads of brush and trees through development and road maintenance, and other logging practices, can sometimes lead to an increase of berry bushes, and this change in abundance or distribution is not linked to climatic changes.

On Haida Gwaii, an ocean micro-nourishment replenishment scheme was implemented in the hopes of mitigating climate change. In the summer of 2012, The Haida Salmon Restoration Corporation dumped “100 tons of iron sulphate, and 20 tons of iron oxide” into the ocean near Old Massett to increase carbon and plankton production, and thus salmon food (Haida Salmon Restoration Corporation 2017). Both RB and A1 (Old Massett) observed that the year after this happened there appeared to be increased numbers of salmon, and they were bigger. However, inputs of this type may disguise the true effects of climate change. While initially this seems like a positive outcome and may be seen as an extension of TEK to new technology, we have yet to learn what the long-term effects are of these types of interventions, and there has been a great amount of controversy over this enrichment programme, including its legality (Abate 2016).

Reg Davidson (Old Massett) noticed that while poles carved 100 years ago had lasted for many decades, more recent poles were getting ‘green’ through vegetation and lichen growing on them relatively soon after they were erected, and they were rotting faster than would be expected. For example, he described this phenomenon “…from the 1800[‘s], there’s one that lasted 100 years before it fell…and now they removed one after what, 40 years”. Thus, because all poles were rotting faster in the last 10–20 years, the new poles weren’t lasting as long as the older poles had. This is possibly an effect of climate change or, as generalized by RD, air pollution. Along a similar vein, in a conservation report of totem poles in Ketchikan, Alaska, Sheetz (2008) noted a particular increase in decay, and of lichen, moss and other vegetation growing out of the totem poles since the last conservation report 14 years previously in 1994, and suggests that this issue of epiphytic growth should be addressed by clearing branches away from poles to increase air circulation, or by applying a fungicide or water repellent compound. This shows that the observation of RD has been seen in other locations as well. Todd (1998) suggests one reason for the totem poles decaying faster now was due to moisture being trapped between the paint and the wood of the pole, as this is a very moist environment. However, this does not explain why there might be more ‘greening’ of the pole now versus in the past, unless this observation can be linked to the increased rain noted overall by participants in this study area.

As we have shown, Pacific Northwest Coast Indigenous Peoples are facing rapidly changing landscape and biodiversity conditions, and this is greatly impacting their traditional lifestyles. Even though store-bought food is common in communities today for daily consumption, the high cost of even basic commodities, and the cultural imperative of maintaining traditions and knowledge through food gathering, mean that local people are still committed to wild food production. Not only do traditional livelihoods immerse and emplace people in their traditional cultural habitats, they are also considered healthier. In many communities, traditional foods are even featured in state-supported social service and health settings, such as senior centres and hospitals. Some interviewees commented that, with the ‘drying up’ of oil, gas and other non-renewable resources, and the rising costs of living in their communities, they would need to depend on the land *more* in the future. To do this, they will have to understand how their ecosystems and habitats are changing and adapt accordingly. The high cost of living traditional lifestyles and the Tlingit relational idea of ‘Social Change’ (social climate change, proposed by KG, Hoonah) also demonstrates how social and physical climate change may exacerbate each other and can lead to a breakdown at which even historically resilient lifeways may become untenable into the future. Issues mentioned by research participants such as fuel costs, needing to travel further to gather resources, the uncertainty in timing of resource availability, and having less time to gather resources in the current economic climate, are indicative of the twofold challenges faced by the Indigenous People in this area.

CKIS can be an important lens through which to improve both understanding of ecosystem changes due to climate change, and help guide potential adaptation strategies to deal with impacts on biodiversity. However, government planning and decision-making processes do not at present focus on TEK or CKIS. Many of our respondents felt that government officials only make token community visits to ask opinions at local meetings, and do not take TEK into consideration in decision-making, or that official harvest rules and regulations are too rigid and standardized to take local knowledge about climate variation into account, or to realize how important CKIS are to local people’s livelihoods and sense of identity. The three policy practitioners interviewed in this study felt that the government was improving its communication with communities, but there are still conceptual and implementation gaps, leading to local knowledge being at best “unevenly incorporated” into climate and resource management policy (LK, Juneau). This uneven or lack of incorporation of local knowledge, in turn, can lead to “skepticism…fear, [and] insecurity… on the part of the Native community to share knowledge” (LK, Juneau). To gain a higher level of conceptual and practical integration of TEK, keystone and indicator species and ecosystem change need to be taken into account in the management of the ecosystem.

However, some co-management successes were noted. Previous research, explicitly mentioned in interviews, documented the importance of TEK in sustainable harvesting of seagull eggs (a CKIS in some island communities) in Glacier Bay (Hunn et al. 2003; CB, KG, Hoonah). Another example of co-management that emerged in the interviews was that of sockeye salmon by the Alaskan government and the Hydaburg Cooperative Association, described by Native leader Anthony Christianson (Hydaburg, see quote in Table 4). Through the community’s TEK of salmon, they were able to identify the specific timings of different runs and collaborate to fine tune regulations to both enhance the monitoring and protection of fish stocks and ensure that local people are still able to harvest salmon under optimal conditions (Cartwright et al. 2005; Conitz et al. 2007). Anthony Christianson received a Community-based Conservation Award in 2015 from The Nature Conservancy for his efforts on this project (Stories in the News 2015). A co-management regime combining TEK and science of the dynamics of these CKIS’ will hopefully generate adaptive solutions to allow this traditional resource to continue to be sustainably harvested, which can also be applied in other communities.

There are additional co-management examples located throughout the study area. The Sustainable Southeast Partnership in Alaska is one such example, bringing together seven Indigenous communities (and includes Hoonah, Hydaburg, Kake, and Klawock from this research) and numerous organizations such as Sealaska, The Nature Conservancy, Southeast Alaska Watershed Coalition, Renewable Energy Alaska Project, Southeast Alaska Regional Health Consortium, and Alaska Conservation Foundation, all of whom coordinate with private, state and federal land managers to manage local fisheries and forests in the best interests of the local communities (Sustainable Southeast Partnership 2019). Another similar example, but in British Columbia, is the Marine Plan Partnership for the North Pacific Coast, bringing together the government of British Columbia and 17 First Nations to design and execute plans for the use of marine resources (Marine Plan Partnership for the North Pacific Coast 2019), divided into four sub-regions. Haida Gwaii is one of these sub-regions, and they have specifically designed the Haida Gwaii Marine Plan to allow the Haida Gwaii Indigenous communities and the province to co-manage the local marine resources (Marine Planning Partnership Initiative 2015). Another example is the several organizations incorporated in the management of fish stocks in the United States as a whole, from a governmental viewpoint. Firstly, the Magnuson-Stevens Fishery Conservation and Management Act (MSA), a law implemented in 1976, was developed with the intention of collaborating with stakeholders to protect and manage fishing resources. In practice, this means that the National Marine Fisheries Service (NMFS, under the auspices for National Oceanic and Atmospheric Administration, NOAA) monitors policy throughout the entire nation, while one of the eight councils forges a link between the government and stakeholders, to input and agree on management decisions (Alaska Marine Conservation Council 2019). Local Alaskan fisheries are monitored by the North Pacific Fishery Management Council (NPFMC), which advises on federal stocks (ground-fish, i.e. cod, pollock, flatfish, mackerel), and jointly monitors, with the State of Alaska, salmon, crab and scallop stocks. One example of where the local government organizations, Indigenous community members, local fisherman and scientists have worked together to manage fish stocks sustainably is around the issue of trawling, which has now been banned parts of Alaska, including in Southeast Alaska, due to its destructiveness to the habitat and high rates of bycatch (Stiles et al. 2010; North Pacific Fishery Management Council 2012; Sitka Conservation Society 2012).

## Conclusion: CKIS species and human adaptation to biodiversity change

It can be seen that Peoples on the Northwest Coast are, and have been, experiencing and adapting to climatic changes through continued interactions with their changing ecosystems and biodiversity over many thousands of years. An emphasis on CKIS provides an opportunity to advance the inclusion of Indigenous knowledge and TEK into climate research, and posit ways of dealing with the totality and complexity of what Indigenous Peoples have to deal with in a meaningful, positive, and empowering way. Understanding human responses to biodiversity change through CKIS also helps move adaptation studies beyond vulnerability-based approaches (Ford and Smit 2004; Ford et al. 2006; Ford 2009; Cameron 2012; Ford et al. 2018) towards strength-based approaches which favour not only local knowledge but culturally important species which are the best sources of understanding the impacts of climate change and important lenses through which to see potential adaptation opportunities. Significant impacts on all sets of CKIS described above are evident as a result of climatic change, while at the same time we find participants responding to these impacts in practical ways, and prioritizing their responses in different ways, albeit with various limitations. For example, while people are able to somewhat adjust how and when they go out fishing due to inclement weather or fish behaviour change, other factors such as holiday time from work or the governmental control of fishing seasons affect how much they can modify their behaviour to prioritize their responses to these new changes. Thus, the results from this research suggest some prioritizing; however more investigations are needed to fully understand the ramifications of how people can prioritize within modern governmental and lifestyle constraints. While we only consider three species/functional groups of CKIS in this brief space, this concept can easily be expanded to include others, which inhabit similar or other landscapes. A programme of CKIS species could be developed as a part of a comprehensive research, monitoring, and evaluation (and perhaps restoration) programme for addressing climate change, and biodiversity and human responses to its impacts, in the Northwest Coast Region. As more research is conducted looking at weather baselines in the Pacific Northwest, and the nature of faster paced changes in the present ‘Anthropocene’ versus earlier epochs, an even better understanding of the intersections and correlations between Indigenous observations of the earth system and climate data can be achieved.

## Electronic supplementary material

Below is the link to the electronic supplementary material.

Supplementary material 1 (PDF 129 kb)
