# Supplementary material for: Using traditional ecological knowledge to understand and adapt to climate and biodiversity change on the Pacific coast of North America
Source: Ambio. 2019 Oct 9;48(12):1447–69. doi: 10.1007/s13280-019-01218-6 (PMC6882747; doi:10.1007/s13280-019-01218-6)
Supplement: Supplementary file 1 — Supplementary material 1 (PDF 129 kb) [file 13280_2019_1218_MOESM1_ESM.pdf]

Ambio

Electronic Supplementary Material

Title: **Using traditional ecological knowledge to understand and adapt to climate and biodiversity change on the Pacific coast of North America**

Authors: Victoria Rawn Wyllie de Echeverria, Thomas F. Thornton

APPENDIX 1. Interviewees, highlighted in light green denotes married couples

Initials	Name	Community	Gender	Age/ age range
DA	Doris Auckland	Metlakatla, BC	Female	93
LB	Lionel Bean	Kake, AK	Male	-
NB	Nancy Bean	Kake, AK	Female	-
AB	Arnie Bellis	Old Massett, BC	Male	61
LB	Lorna Berekoff	Skidegate, BC	Female	-
CB	Catherine J. Bolton	Hoonah, AK	Female	30s-40s
DB	Daniel W Bolton	Hoonah, AK	Male	30s-40s
DBr	Diane Brown	Skidegate, BC	Female	-
RB	William R (Robin) Brown	Old Massett, BC	Male	81
WB	Wilbur G. Brown, Sr.	Kake, AK	Male	81
AC	Anthony Christianson	Hydaburg, AK	Male	37
DC	Delores Churchill	Ketchikan, AK; family from Old Massett, BC (and interviewed there)	Female	-
HC	Helen Clifton	Hartley Bay, BC	Female	80s
ACo	Alma P. Cook	Klawock, AK	Female	90
WC	Wanda J. Culp	Hoonah, AK	Female	66
RD	Reg Davidson	Old Massett, BC	Male	60
GD	George L. Davis	Juneau, AK (from Kake)	Male	-
AD	Agnes Davis	Juneau, AK (from Kake)	Female	-
JD	June Degnan	Juneau, AK	Female	-
RDe	Ruth Demmert	Kake, AK	Female	77
SD	Sherri Dick	Old Massett, BC	Female	56
GE	Gwaii Edenshaw	Old Massett, BC	Male	-
ME	Margaret Edgars	Old Massett, BC	Female	-
LG	Leo Gagnon	Old Massett, BC	Male	-
CG	Captain Gold [Richard Solomon Wilson]	Skidegate, BC	Male	-
KG	Kenneth J. Grant	Hoonah, AK	Male	Over 70
AG	Adam Greenwald	Hoonah, AK	Male	87
EG	Ester Greenwald	Hoonah, AK	Female	-
LKG	Lori Kay Guinard	Hoonah, AK	Female	52
MG	Mary E. Guthrie	Klawock, AK	Female	65
FH	Fred Hamilton	Craig, AK	Male	-
EHA	Earnestine Hanlon Abel	Hoonah, AK	Female	-
JH	John Hillman	Hoonah, AK	Male	-

MH	Merle Nancy Hawkins	Ketchikan, AK	Female	55
CJ	Charles Jack Jr.	Hoonah, BC	Male	-
JJ	Joel Jackson	Kake, AK	Male	58
MJ	Michael Jackson	Kake, AK	Male	60
OJ	Owen James	Hoonah, AK	Male	-
MJo	Marlene Johnson	Juneau, AK (from Hoonah)	Female	-
CJo	Cliff Johnson	Juneau, AK	Male	-
SJ	Sphenia Jones	Old Massett, BC	Female	
CoJo	Cora Joseph	Klawock, AK	Female	71
EK	Evans Kadake	Kake, AK	Male	78
MK	Marvin Kadake	Kake, AK	Male	-
EKa	Ernestine Kato	Klawock, AK	Female	75
VLC	Vickie Le Cornu	Hydaburg, AK	Female	-
JL	Jack Litrell	Old Massett, BC	Male	-
HM	Harold Martin	Juneau, AK (from Kake)	Male	80
JM	Jackie Martin	Juneau, AK (from Kake)	Female	-
JMa	James Martinez	Klawock, AK	Male	81
RM	Rosa Miller	Juneau, AK	Female	-
AM	Alice Montjoy	Old Massett, BC	Female	-
JMo	John Morris	Juneau, AK	Male	
CN	Charles N. Natkong	Hydaburg, AK	Male	-
FN	Fanny Nelson	Metlakatla, BC	Female	-
RN	Robert Nelson	Metlakatla, BC	Male	-
MOl	Marie Olson	Juneau, AK	Female	-
MO	Margaret O’Neil	Hydaburg, AK	Female	61
TP	Theodore O. Peale	Hydaburg, AK	Male	54
PP	Pearle Pearson	Skidegate, BC	Female	-
OP	Olive Pollard	Skidegate, BC	Female	-
BR	Betty Richardson	Skidegate, BC	Female	-
CR	Crystal Robinson	Old Massett, BC	Female	-
JR	June Russ	Old Massett, BC	Female	-
MR	May Russ	Old Massett, BC	Female	-
TR	Teresa Russ	Old Massett, BC	Female	-
CRy1	Charlene Ryan	Metlakatla, BC	Female	-
CRy2	Clifford Ryan	Metlakatla, BC	Male	-
LS	Larry Sanders	Hoonah, AK	Male	-
FS	Frances Sanderson	Hydaburg, AK	Female	86
RS	Robert Sanderson	Hydaburg, AK	Male	79
MES	Mary Ellen Skinna	Klawock, AK	Female	-
GS	Goldie Swanson	Old Massett, BC	Female	-
MS	Mary Swanson	Old Massett, BC	Female	-
GW	Geraldine P. Wallace	Hoonah, AK	Female	-
EW	Emily Watts	Old Massett, BC	Female	-

CW	Christian White	Old Massett, BC	Male	-
CWi	Sandra (Cindy) Williams	Skidegate, BC	Female	-
HWi	Harriet Williams	Kake, AK	Female	84
HW	Harvey Williams	Skidegate, BC	Male	-
RW	Rolly Williams	Old Massett, BC	Male	53
MY	Myrna Yates	Craig, AK	Female	-
CY	Carol Young	Skidegate, BC	Female	-
Anonymous people				
A1		Old Massett, BC	Male	-
A2		Old Massett, BC	Male	-
A3		Skidegate, BC	Male	-
A4		Hoonah, AK	Female	-
A5		Skidegate, BC	Female	50s
A6		Old Massett, BC	Female	25
A7		Old Massett, BC	Female	-
A8		Ketchikan, AK	Male	-
A9		Juneau, AK (from Kake)	Male	-
A10		Hoonah, AK	Female	72

Government people

Initials	Name	Location	Agency
TF	TerryLee Fiske	Hoonah, AK	US Forest Service
LK	Linda Kruger	Juneau, AK	US Forest Service
MBM	Mary Beth Moss	Hoonah, AK	US Forest Service
